# Conversion of CD73^hi^FR4^hi^ anergic T cells to IFN-γ–producing effector cells disrupts established immune tolerance

**DOI:** 10.1172/JCI163872

**Published:** 2023-03-01

**Authors:** Anil Dangi, Irma Husain, Collin Z. Jordan, Shuangjin Yu, Xunrong Luo

**Affiliations:** 1 Nephrology, Duke University Medical Center, Durham, North Carolina, USA.; 2 Organ Transplantation, First Affiliated Hospital of Sun Yat-Sen University, Guangzhou, China.

**Keywords:** Immunology, Transplantation, Anergy, Cellular immune response, Tolerance

**To the editor:** Anergic T (T_AN_) cells marked by CD73^hi^FR4^hi^ have been shown to differentiate to immunosuppressive populations, such as FoxP3^+^ Tregs or IL-10–producing Tr1 cells (1, 2), and are therefore deemed harmless to stable immune tolerance. However, their potential to differentiate to pathological IFN-γ–producing effector cells has not been studied. We developed an allogeneic transplant tolerance model to investigate this possibility.

We used a BALB/c-to-C57BL/6 (B6) islet transplant model. Donor-specific transplant tolerance was induced in recipients by infusing on days –7 and +1 donor splenocytes treated ex vivo with ethylenecarbodiimide (3) and has been indefinitely maintained in unmanipulated recipients as described previously (4). We first examined the presence of CD73^hi^FR4^hi^ T_AN_ cells in tolerized recipients. As shown in Figure 1A, the majority of intragraft CD44^+^FoxP3^–^ CD4^+^ T cells were CD73^hi^FR4^hi^ T_AN_ cells. These cells were also present in the spleens of tolerized recipients, albeit less prominently than in allografts. In contrast, there was a significantly smaller T_AN_ population in the spleens of nontolerized mice.

We next perturbed this stable tolerance on day 95 after transplantation by giving acute murine cytomegalovirus (MCMV) infection to tolerized mice, which has been previously shown to precipitate allograft rejection in approximately 60%–70% recipients over the ensuing 5–6 weeks (5). Interestingly, at the time of rejection, we observed a significant reduction of the number of intragraft CD73^hi^FR4^hi^ T_AN_ cells, along with a significantly reduced level of CD73 and FR4 expression on remaining T_AN_ cells (Figure 1B). We confirmed that intragraft FoxP3^+^ Tregs, known to similarly express CD73 and FR4, continued to exhibit the same level of CD73 and FR4 before and after MCMV infection (Supplemental Figure 1; supplemental material available online with this article; https://doi.org/10.1172/JCI163872DS1).

Next, we determined the fate of CD73^hi^FR4^hi^ T_AN_ cells in response to MCMV infection. In vitro, we FACS-sorted T_AN_ cells (CD3^+^CD4^+^CD44^+^CD25^–^CD73^hi^FR4^hi^) from spleens of tolerized B6 mice (verified to be indeed anergic; Supplemental Figure 2). We cultured them for 5 days with B6 bone marrow–derived DCs with and without MCMV pretreatment and with and without pulse with BALB/c cell lysate (Supplemental Methods). As shown in Figure 1C, T_AN_ cells cocultured with MCMV-infected DCs with and without pulse with BALB/c lysates showed a marked downregulation of CD73 and FR4 (*P* < 0.05), acquired cell surface CD25 expression (data not shown), and began to prominently express the Th1 cytokine IFN-γ among the proliferating (Ki-67^+^) subset. Interestingly, LPS-treated DCs, in contrast to MCMV-treated DCs, did not lead to any downregulation of CD73 or FR4 on T_AN_ cells (Supplemental Figure 3). In vivo, we similarly FACS-sorted T_AN_ cells for CD45.2 mice and transferred them to CD45.1 mice, followed by MCMV infection a day later. As shown in Figure 1D, 1 week after MCMV infection, a substantial portion of the CD45.2^+^ T_AN_ cells became CD73^–^FR4^–^ and began to produce IFN-γ; in contrast, without MCMV infection, the CD45.2^+^ T_AN_ cells remained CD73^hi^FR4^hi^. Collectively, these data support that MCMV infection reverts the anergic phenotype of T_AN_ cells, likely via DCs, and promotes their differentiation to IFN-γ–producing cells.

Finally, we determined the functional significance of T_AN_ cells in MCMV-mediated disruption of stable tolerance. First, we depleted T_AN_ cells in stably tolerized recipients with a course of anti-FR4 (clone TH6) from day 95 to 120 (100 μg i.v. every 5 days for 6 doses), followed by MCMV infection on day 125. Using a different clone of anti-FR4 (12A5), we confirmed that this course of anti-FR4 indeed effectively depleted CD4^+^FR4^+^ but not other cells (Supplemental Figure 4). As shown in Figure 1E, left, none of the recipients treated with anti-FR4 experienced allograft rejection (followed up to ~day 150). Furthermore, this treatment with anti-FR4 prior to MCMV infection completely prevented MCMV-precipitated rejection in previously tolerized recipients (Figure 1E, right).

To further corroborate above findings from the anti-FR4 experiment, we adoptively transferred sorted T_AN_ cells to B6.RAG^–/–^ mice bearing BALB/c islets, followed by MCMV infection and infusion of naive CD8 T cells (Figure 1F, experimental scheme). We first observed that T_AN_ cells converted to CD73^–^FR4^–^ T cells following MCMV infection (Figure 1G). In addition, mice receiving T_AN_ cells rejected the BALB/c islet allograft following MCMV infection and naive CD8 T cell infusion, whereas mice not receiving T_AN_ cells did not, despite identical MCMV infection and naive CD8 T cell infusion subsequently (Figure 1H). These data substantiate that the T_AN_ cells are crucial in driving rejection in MCMV-infected mice.

Findings in this study refute the notion that CD73^+^FR4^+^ T_AN_ cells are simply passive cells, innocently present during immune tolerance, and instead support that, when appropriately stimulated, these cells can differentiate to IFN-γ–producing Th1 cells to promote immunity. More importantly, we developed a therapeutic strategy for preserving the stability of tolerance by preemptively depleting T_AN_ cells prior to immune perturbation. In our model, the CD73^+^FR4^+^ cells expressed lower levels of the inhibitory molecules CTLA4 and TIGIT in comparison to FoxP3^+^ Tregs (data not shown), suggesting cell-intrinsic factors that determine their fate in response to external stimuli. Our data also point to cell-extrinsic factors originating from partners that interact with T_AN_ cells, specifically DCs, that play a critical role in their differentiation to effector cells. Collectively, our findings underscore the potential detriment of anergic T cells, which may seem benign, in tolerant recipients. Additionally, we support the efforts of future studies to identify crucial elements of anergic T cell instability and therapeutic targets to prevent their differentiation to proinflammatory cells.

## Supplementary Material

Supplemental data

## Figures and Tables

**Figure 1:**
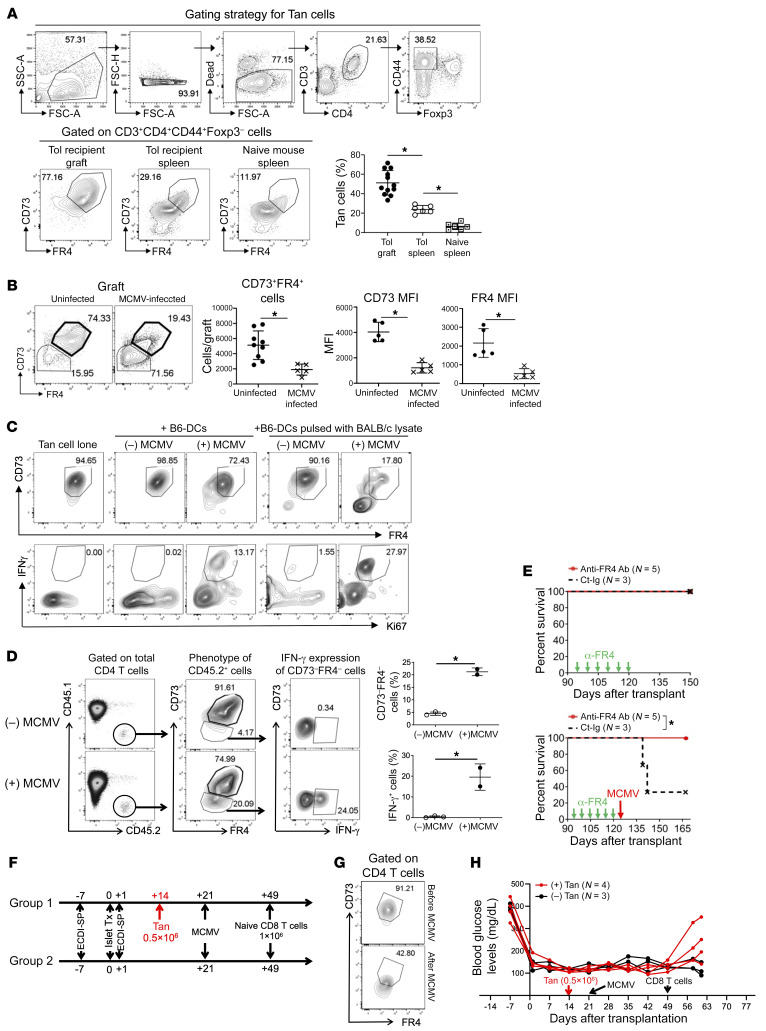
Conversion of CD73^hi^FR4^hi^ anergic T cells to IFN-γ–producing cells disrupts established transplantation tolerance. (**A**) T_AN_ cells are enriched in islet allografts and spleens of tolerized recipients. *n* = 6–12 per group. (**B**) Loss of T_AN_ cells in islet allografts following MCMV infection of stably tolerized recipients. *n* = 3–9 per group. (**C**) T_AN_ cells from tolerized recipients are induced to produce IFN-γ by MCMV-infected DCs (representative of 2 independent experiments). (**D**) In vivo conversion of adoptively transferred CD45.2 T_AN_ cells following MCMV infection of CD45.1 hosts. *n* = 2–3 per group. (**E**) Depletion of T_AN_ cells prevents MCMV-mediated transplant tolerance disruption. *n* = 3–5 per group. (**F**) Experimental scheme in RAG^–/–^ mice. (**G**) In vivo conversion of adoptively transferred T_AN_ cells following MCMV infection of RAG^–/–^ (representative of *n* = 4). (**H**) Rejection of islet allografts in RAG^–/–^ following T_AN_ adoptive transfer. *n* = 3–4 per group. *P < 0.05. (**A**–**D**) Mann-Whitney test. (**E**) Log-rank test.

## Version 1 01/17/2023

In-Press Preview

## Version 2 03/01/2023

Electronic publication
