# Supplementary material for: Conversion of CD73hiFR4hi anergic T cells to IFN-γ–producing effector cells disrupts established immune tolerance
Source: J Clin Invest. 2023 Mar 1;133(5):e163872. doi: 10.1172/JCI163872 (PMC9974094; doi:10.1172/JCI163872)
Supplement: Supplemental data [file jci-133-163872-s102.pdf]

**Conversion of $CD73^{hi}FR4^{hi}$ anergic T cells to IFN-$\gamma$-producing effector cells disrupts established immune tolerance**

**Supplemental Materials**

**Supplemental Method** ……………………………………………………………………...2

**Supplemental Figure 1.** MCMV infection does not alter the expression level of CD73 or FR4 on intragraft $CD4^+FoxP3^+$ $T_{REG}$ cells …………………………………………………....4

**Supplemental Figure 2**. $CD73^{hi}FR4^{hi}$ cells from tolerized B6 mice are indeed anergic ……………………………………………………………………………………………….5

**Supplemental Figure 3**. LPS treated DCs do not down-regulate CD73 or FR4 on $T_{AN}$ cells following DC-T co-cultures ………………………………………………………………6

**Supplemental Figure 4**. Anti-FR4 (clone TH6) depletion pattern *in vivo* ………………..7

## Supplemental Methods

### Animals

Allogeneic islet transplantation was performed using the kidney sub-capsular site as we described before. Blood glucose (BG) levels were used to follow islet allograft function post transplantation. BG levels >= 250mg/dL on two consecutive days were used to indicate rejection of the islet allograft.

### Study approval

All procedures involving mouse studies were reviewed and approved by Duke Institutional Animal Care and Use Committee (IACUC) located in Durham, North Carolina. The approved Duke University IACUC protocol number is A215-21-10.

### MCMV infection

For *in vitro* MCMV infection, DCs were pre-treated with MCMV at a multiplicity of infection (MOI) of 1 for two days followed by removal of the virus before co-culturing the DCs with $T_{AN}$ cells. For *in vivo* MCMV infection, in immune competent mice, $10^7$ PFU of MCMV was given via one intraperitoneal injection. In $RAG^{-/-}$ mice, $10^5$ PFU of MCMV was given via one intraperitoneal injection.

### DC and $T_{AN}$ cell co-cultures

DCs were co-cultured with $T_{AN}$ cells at a 3:1 ratio in 96 well plates for 5 days before harvesting for T cell analysis. When indicated, BALB/c lysate was used to pulse B6 DCs at a final concentration of 50μg/mL for 3-4 hours before $T_{AN}$ cells were added. When

indicated, LPS at a final concentration of 100ng/mL was used to activate B6 DCs for two days followed by its removal before co-culturing with $T_{AN}$ cells.

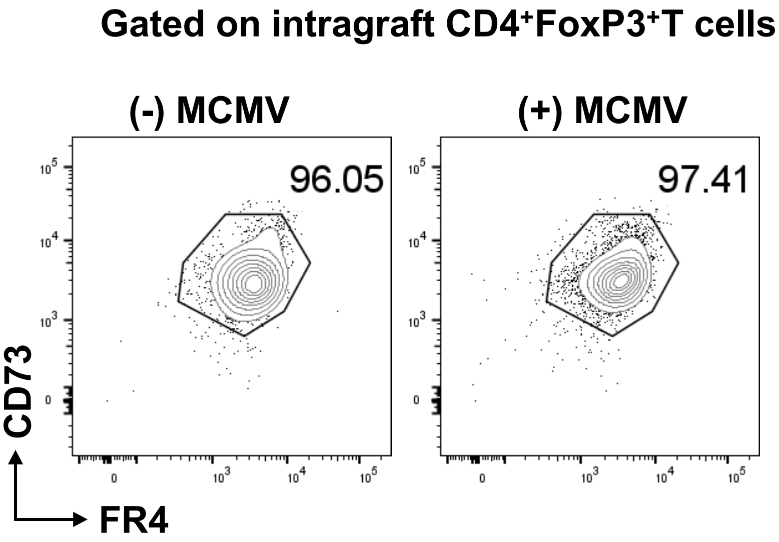


**Supplemental Figure 1. MCMV infection does not alter the expression level of CD73 or FR4 on intragraft CD4$^+$FoxP3$^+$ $T_{REG}$ cells.** Data presented is representative of N=8.

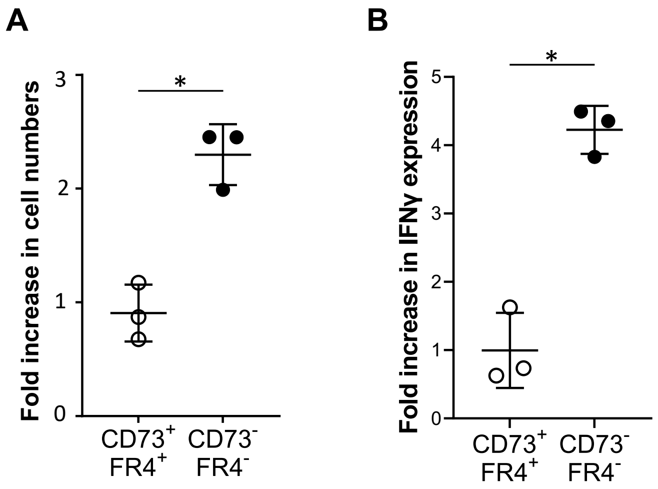


**Supplemental Figure 2**. **CD73$^{hi}$FR4$^{hi}$ cells from tolerized B6 mice are indeed anergic.** CD44$^{+}$CD73$^{hi}$FR4$^{hi}$ cells and CD44$^{+}$CD73$^{-}$FR4$^{-}$ cells from tolerized B6 mice were stimulated with anti-CD3/CD28 beads for 3 days, and proliferation (**A**) and IFN-γ production (**B**) were measured. Comparisons were made to baseline. *$P$<0.05.

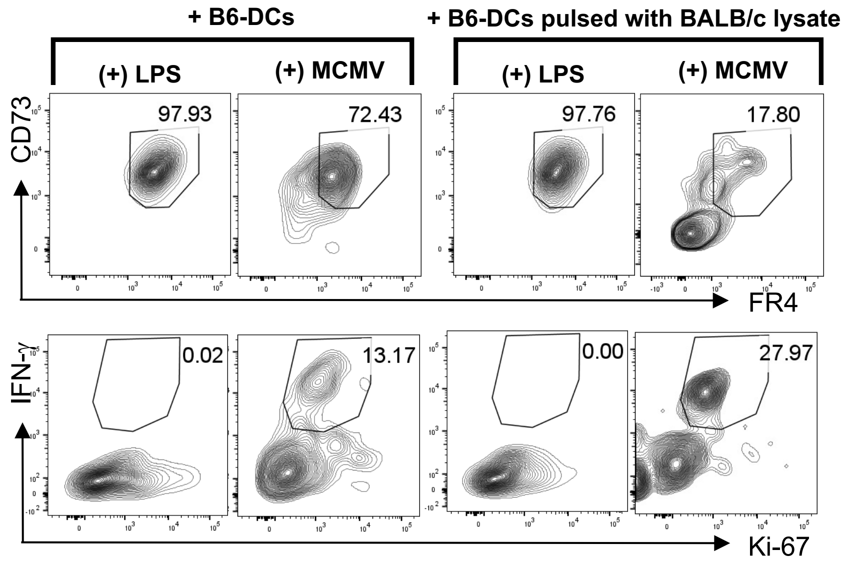


**Supplemental Figure 3. LPS-treated DCs do not down-regulate CD73 or FR4 on $T_{AN}$ cells following DC-T co-cultures.** $T_{AN}$ cells from tolerized recipients do not lose anergy markers or produce IFN-γ when co-cultured with LPS-treated DCs, in contrast to when con-cultured with MCMV-infected DCs (representative of 2 independent experiments).

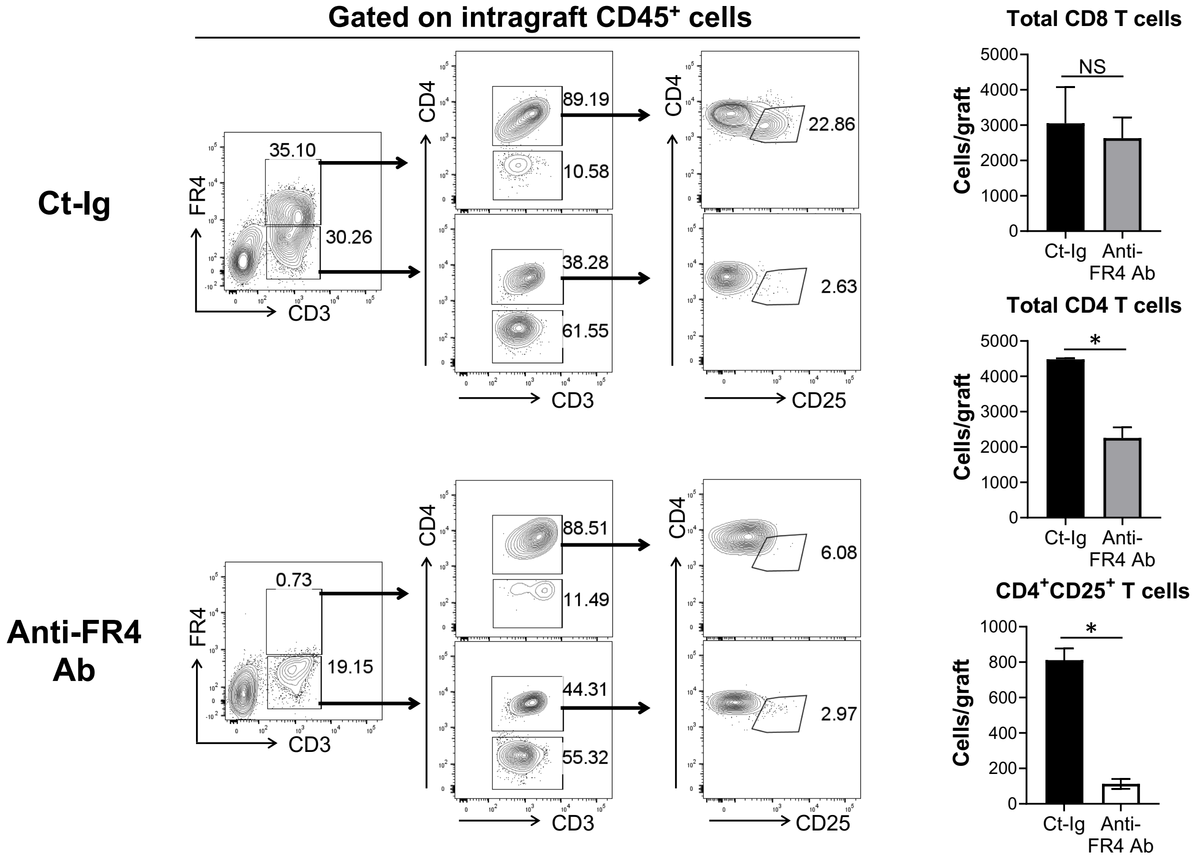


**Supplemental Figure 4**. **Anti-FR4 (clone TH6) depletion pattern *in vivo*.** Islet grafts were collected following treatment with anti-FR4 (clone TH6) or control Ig (Ct-Ig), and stained with indicated markers as well as FR4 (with clone 12A5). (**A**) Representative FACS plots (representative of N=3-5 per group). (**B**) Total numbers of graft $CD8^+$, $CD4^+$ and $CD4^+CD25^+$ T cells. N=3-5 per group. *$P$<0.05.
